# Independent Association between Nonalcoholic Fatty Liver Disease and Cardiovascular Disease: A Systematic Review and Meta-Analysis

**DOI:** 10.1155/2013/124958

**Published:** 2013-04-10

**Authors:** Hongyun Lu, Hong Liu, Fang Hu, Lingling Zou, Shunkui Luo, Liao Sun

**Affiliations:** Department of Endocrinology & Metabolism, The Fifth Affiliated Hospital of Sun Yat-sen University, Zhuhai, Guangdong 519000, China

## Abstract

Nonalcoholic fatty liver disease (NAFLD) is closely correlated with insulin resistance and several metabolic syndrome features, but whether it could increase the risk of cardiovascular disease remains undefined. To assess the association between NAFLD and the risk of cardiovascular outcomes, we systematically searched the MEDLINE, Embase, and the Cochrane Library database (1947 to October 2012) by using Medical Subject Heading search terms and a standardized protocol. Randomized controlled trials, case-control, and prospective studies carried out in human adults, in which the unadjusted and multivariate adjusted odds ratios with corresponding 95% confidence interval (CI) for cardiovascular disease with NAFLD were reported. The search yielded 4 cross-sectional studies and 2 prospective cohort studies including 7,042 participants. The pooled effects estimate showed that NAFLD was a predictor of cardiovascular disease (odds ratio 1.88, 95% CI, 1.68 to 2.01; *P* < 0.001). The random effects summary estimate indicated that NAFLD retained a significant association with cardiovascular outcomes independent of conventional risk factors after adjustment for established cardiovascular risk factors (odds ratio 1.50, 95% CI, 1.21 to 1.87; *P* < 0.001). These results indicate that NAFLD is a strong independent predictor of cardiovascular disease and may play a central role in the cardiovascular risk of metabolic syndrome.

## 1. Introduction

 Nonalcoholic fatty liver disease (NAFLD) represents a wide spectrum of hepatic disorders in clinical practice [1], the prevalence in the general population is 10%~30%, and the number increases greatly whether in developing or developed countries [2–5]. It has been convincingly associated with insulin resistance and metabolic syndrome (MS); most patients are overweight or frankly obese, with altered glucose metabolism, dyslipidemia, and raised blood pressure, all contributing to the disorders [6–8].

However, the clinical and public health significance of NAFLD is not well established. People with NAFLD harbor the same cardiovascular risk factors (hypertension, dyslipidaemia, obesity, physical inactivity, insulin resistance, endothelial dysfunction, and inflammation) that place them at high risk of cardiovascular events [9, 10]. Recently, some studies showed that subjects with NAFLD have an elevated risk of increased carotid intima media thickness [11, 12], reduced endothelial function [13], increased coronary artery calcification [14, 15], and increased arterial stiffness [16]. However, other studies indicated that NAFLD was not associated with MS and cardiovascular disease [17]. Despite these results, it remained controversial whether NAFLD was a marker or an independent mediator that promotes cardiovascular disease, and the effect of NAFLD on the risk of future cardiovascular events has not been well established.

Hence, we performed a systematic review and meta-analysis with the most updated prospective data to evaluate the association of NAFLD with the risk of incident cardiovascular outcomes in patients.

## 2. Methods

 The search strategy was in accordance with the recommendations of the meta-analysis of observational studies in epidemiology (MOOSE) group. We searched EMBASE (1947 to October 16, 2012), MEDLINE (1947 to October 16, 2012), The Cochrane Library (1947 to October 16, 2012), Science Citation Index (Web of Knowledge) (1947 to October 16, 2012), and PubMed (1947 to October 16, 2012), using the search terms “nonalcoholic fatty liver disease” or “NAFLD” or “fatty liver” AND “cardiovascular disease” or “myocardial ischemia” or “MI” or “myocardial infarct” or “ischemic heart disease” or “coronary heart disease” or “coronary artery disease” or “angina” or “stroke” or “cerebrovascular disease” or “cerebrovascular attack” or “cerebral ischemia” or “brain ischemia” or “intracranial hemorrhage.” The search had no language restriction.

### 2.1. Inclusion and Exclusion Criteria

An article was considered relevant if it reported quantitative estimates of the unadjusted and (or) multivariable adjusted (i.e., age, sex, smoking history, diabetes duration, HbA1c, LDL cholesterol, GGT (gamma-glutamyl transpeptidase) levels, and use of medications (i.e., hypoglycemic, antihypertensive, lipid-lowering, or antiplatelet drugs), or additional adjustment for the presence of metabolic syndrome and/or NAFLD) odds ratio with corresponding 95% confidence interval (CI) for the log relative risk for cardiovascular events. NAFLD patients were evaluated at least by abdominal ultrasound or computed tomography (CT). Cardiovascular events include coronary heart disease (such as myocardial infarction, angina pectoris, and ischemic stroke), cerebrovascular disease (such as cerebral hemorrhage), and peripheral vascular disease. The criteria of National Cholesterol Education Program Adult Treatment Panel III (NCEP ATP III) are used to characterize the metabolic syndrome (MS) [18]. Unpublished papers, nonhuman studies, letters/case reports, studies enrolling <10 subjects or subjects aged <12 years, editorials, reviews, no cardiovascular endpoints, no multivariate adjusted cardiovascular events estimate, or using inadequate case definition were excluded.

### 2.2. Data Extraction and Quality Assessment

The data extraction was performed independently by two authors and included first author, date of publication, location of research group, number of analyses, characteristics of participants, study design, outcome measures, and selection criteria. We extracted the unadjusted and (or) multivariable adjusted odds ratio for cardiovascular events and corresponding 95% CI in the statistical analysis. Reference lists of the retrieved articles were searched for additional publications. The quality of the selected studies was assessed independently by two authors using the Newcastle-Ottawa Scale (NOS) for cohort and cross-sectional studies. The NOS used a “star” rating system to judge quality based on three aspects of the study: selection of study groups, comparability of study groups, and ascertainment of either the exposure or outcome of interest [19]. Any discrepancies were addressed by a joint reevaluation of the original article with another author.

### 2.3. Statistical Analysis

The results of each study were reported as an odds ratio. To measure the outcome, the DerSimonian-Laird method and random-effects model (REM) were used. Results were expressed as pooled odds ratios (OR (95% confidence intervals, CIs)). We assessed whether a significant level of difference existed using Mantel-Haenszel chi-square tests. If the chi square test was significant below *P* = 0.05, we quantified the amount of heterogeneity using *I* ^2^ statistics. We considered *I* ^2^ above 50% as indicative of substantial heterogeneity [20]. The potential for publication bias was addressed by drawing funnel plots and visual assessment [21]. Meta-analyses were performed for unadjusted and multivariate adjusted cardiovascular events estimate with NAFLD and MS, separately. We used the Stata10 software package for the meta-analysis of observational studies. In the forest plots, OR values > 1 represent a direct association and <1 an inverse association. The size of the squares was correlated with the weight of the respective study [21].

## 3. Results

### 3.1. Search Results

**Figure 1:**
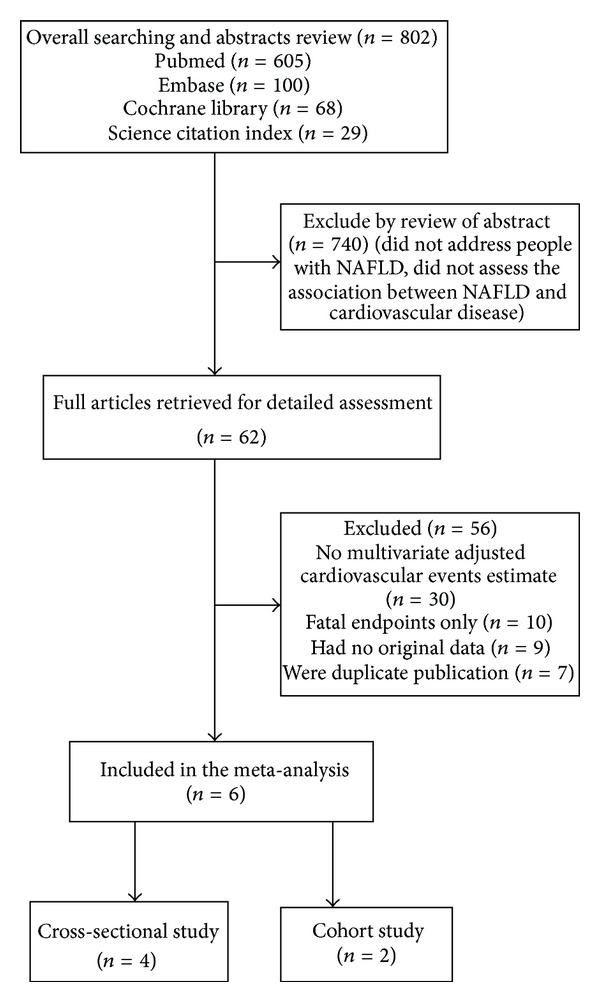
Flow diagram of studies assessed and included.

The literature search yielded a total of 802 potentially relevant abstracts. Seven hundred and forty articles were excluded by review of abstract because they did not address people with NAFLD, or they did not assess the association between NAFLD and cardiovascular disease. By reviewing full articles, 56 articles were rejected because they (1) have no multivariate adjusted cardiovascular events estimate, (2) fatal endpoints only, (3) had no original data (review, editorials), or (4) were duplicate publications. Finally, six studies (Figure 1) were included in our analysis [8, 22–26]. 

### 3.2. Characteristics of the Studies

 The main characteristics of the studies included in this analysis are provided in Table 1. Among them, one study originated from Korea, one from Israel, three from Italy (they were conducted by the same research group among diabetics, but three studies did not include the same participants), and one from Japan, a total of 7,042 participants. According to the NOS score, the six studies were of high quality.

### 3.3. Outcome Results

**Figure 2:**
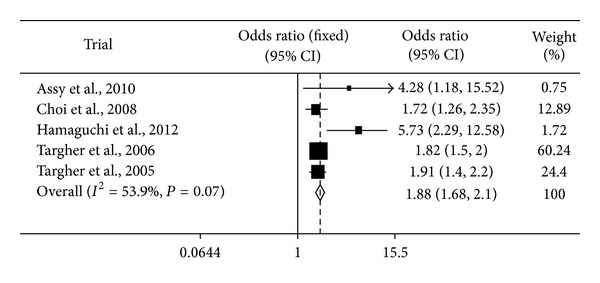
Summary estimates for Mantel-Haenszel odds ratios, the corresponding 95% CI limits, and significance (*P* value) were estimated by fixed effects metaregression analysis for cardiovascular disease between the two groups (NAFLD patients and controls). In the graph, numbers indicate OR values, filled squares stand for the effect of individual studies, and the diamond expresses combined fixed effects.

These six studies all reported adjusted odds ratio with corresponding 95% CI, which allowed us to pool their data into a further analysis.  Figure 2 shows a univariate meta-analysis unadjusted odds ratio [24] for cardiovascular disease with NAFLD. The analyses were based on the fixed-effect model. Heterogeneity chi-square = 8.67, d.f. = 4, and *P* = 0.07. We did not find significant evidence of heterogeneity across studies (*P* for heterogeneity > 0.05). The fixed effects summary estimate shows an increased risk of cardiovascular disease (odds ratio 1.88, 95% confidence interval 1.68 to 2.10; *P* < 0.001) and no major asymmetrical appearance in the funnel plot.

**Figure 3:**
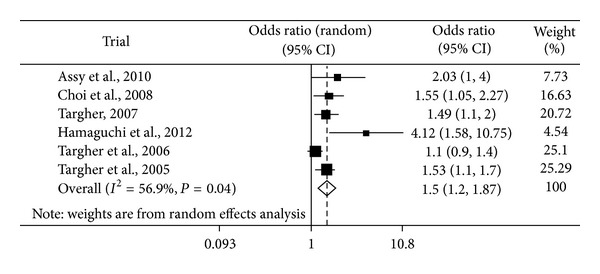
Summary estimates (after adjustment for confounders) for Mantel-Haenszel odds ratios, the corresponding 95% CI limits, and significance (*P* value) were estimated by random effects metaregression analysis for cardiovascular disease with NAFLD patients.

Thus, we did a multivariable adjusted odds ratio meta-analysis based on the primary outcome reported in all studies for cardiovascular disease with NAFLD. The analyses were based on the random-effect model and are presented in Figure 3. Heterogeneity chi-square = 11.61 (d.f. = 5), *P* = 0.04, *I*-squared = 56.9%, and *τ*2 = 0.0373. The random effects summary estimate shows an increased risk of cardiovascular disease after adjustment for established cardiovascular risk factors (age, sex, diabetes duration, HbA1c, smoking history, LDL cholesterol, GGT levels and use of medications (i.e., hypoglycemic, antihypertensive, lipid-lowering or antiplatelet drugs) and NCEP ATP III-defined MS) (odds ratio 1.50, 95% CI 1.21 to 1.87; *P* < 0.001). We found evidence of heterogeneity across studies (*P* for heterogeneity < 0.05) but no major asymmetrical appearance in the funnel plot.

We also did meta-analysis stratifying by study type for cross-sectional and prospective studies based on the primary outcome reported in five studies. The heterogeneity between cross-sectional studies chi-square = 1.82, df = 2, *P* = 0.403, and *I*-squared = 0.0%. We did not find significant evidence of heterogeneity across cross-sectional studies (*P* for heterogeneity > 0.05). The subgroup summary estimate shows increased risk of cardiovascular disease (odds ratio 1.82, 95% CI 1.60 to 2.07; *P* < 0.001) and no major asymmetrical appearance in the funnel plot. The heterogeneity between the prospective studies chi-square = 5.97, df = 1, *P* = 0.015, and *I*-squared = 83.3%. We found significant evidence of heterogeneity across prospective studies (*P* < 0.05). The subgroup summary estimate shows increased risk of cardiovascular disease (odds ratio 2.05, 95% CI 1.65 to 2.55; *P* < 0.001) and no major asymmetrical appearance in the funnel plot. We also did meta-analysis based on half of the studies only included diabetic patients. The heterogeneity chi-square = 4.98, df = 2, *P* = 0.083, and *I*-squared = 59.8%. We did not find significant evidence of heterogeneity across studies (*P* > 0.05). The estimate shows an increased risk of cardiovascular disease (odds ratio 1.34, 95% CI 1.17 to 1.54; *P* < 0.001) and no major asymmetrical appearance in the funnel plot.

## 4. Discussion

NAFLD is a hepatic manifestation of the metabolic syndrome, it is closely related to other clinical features of the metabolic syndrome, and thus cardiovascular disease is increased in NAFLD and represents the main cause of death in these patients. However, given the shared features between NAFLD, the metabolic syndrome, and traditional cardiovascular risk factors, it remains uncertain whether NAFLD is an independent risk factor for increased cardiovascular event [22, 25, 26]. Several previous studies have demonstrated that patients with NAFLD have significantly higher rates of prevalent coronary, cerebrovascular, and peripheral vascular disease than their counterparts without NAFLD [9–11, 27–31]. However, the lack of diagnostic uniformity and difficulty in accurately quantifying the severity of NAFLD in the various published studies make interpretation of the results challenging and sometimes contradictory [32, 33]. There is an urgent need to ensure a more homogeneous evaluation of study outcomes [34, 35]. To provide a more objective basis for clinical recommendations, we conducted a meta-analysis, which recruited a total of 7,042 individuals from 2 prospective and 4 cross-sectional studies. To our knowledge, this is the first meta-analysis on this topic that provides a complete analysis of the potentially harmful role of NAFLD on cardiovascular disease. In our previous cross-sectional study which was conducted among 560 cases of in-patients type 2 diabetes mellitus patients from January 2002 to January 2009 in Southern China, we found that NAFLD was associated with a higher prevalence of coronary heart disease in type 2 diabetes, and that plasma ALT levels may act as a marker [29]. In this meta-analysis, we confirmed previous data demonstrating the high prevalence of cardiovascular disease in NAFLD patients [14]. More importantly, our findings extend the work of recent small studies showing that patients with NAFLD, as assessed by ultrasonography or computed tomography (CT), had a significant association with cardiovascular mortality.

In our analysis, we found that NAFLD was a predictor of cardiovascular events (pooled univariate odds ratio 1.88, 95% CI 1.68 to 2.10; *P* < 0.001). Even after adjustment for confounders (age, sex, diabetes duration, HbA1c, smoking history, LDL cholesterol, GGT levels and use of medications (i.e., hypoglycemic, antihypertensive, lipid-lowering or antiplatelet drugs), and NCEP ATP III-defined MS), the association remained significant (pooled multivariate odds ratio 1.50, 95% CI 1.21 to 1.87; *P* < 0.001). This analysis shows that NAFLD is an independent novel predictor for cardiovascular events, even when other components of the metabolic syndrome are taken into account. Because of the link between the two disorders, and that the majority of patients diagnosed with NAFLD are asymptomatic [36], more careful surveillance of these patients will be needed [37]. Healthcare providers should recognize this higher risk of cardiovascular disease. Patients should be educated as it is our experience that they become singularly focused on liver enzymes and ignore more important cardiovascular health [35, 38, 39]. All NAFLD patients should be evaluated for their metabolic, cardiovascular, and liver-related risk.

## 5. Strengths and Limitations of Study

Our study has several limitations. Firstly, similar to other reports [9, 16, 33, 40], the diagnosis of NAFLD obtained in our study was based on ultrasonography or computed tomography (CT) and the exclusion of known causes of chronic liver disease but was not confirmed by liver biopsy. Although liver biopsy remains the gold standard for NAFLD diagnosis and evaluation, it is difficult to conduct in large populations, and ultrasonography remains the most common way of diagnosing NAFLD in clinical practice due to its good sensitivity and specificity in detecting moderate and severe steatosis [41]. Secondly, NAFLD ranges from simple steatosis (SS) to nonalcoholic steatohepatitis (NASH) [1, 16, 30, 37]. One recent meta-analysis showed that compared to SS, NASH has a higher liver-related (OR for NASH: 5.71, 2.31–14.13; OR for NASH with advanced fibrosis: 10.06, 4.35–23.25) but not cardiovascular mortality (OR: 0.91, 0.42–1.98) [30]. In our study, we did not take the NAFLD histological subtypes into account.

Despite these limitations, our study also has notable strengths. First, this analysis was obtained by pooling data from a number of clinical trials; the heterogeneity between studies was less evident, which significantly increased the statistical power of the analysis compared to a single study. Second, the quality of studies included in the current meta-analysis was based on the NOS. All of them were of high quality. Third, the included studies originated from different countries and included a variety of ethnic backgrounds, allowing for the generalization of our results. Finally, because this meta-analysis was based on unadjusted and multivariate adjusted estimates, separately, the results of it are possibly the most precise estimate available of the strength of the relation between NAFLD and future risk of cardiovascular events.

In conclusion, our results suggest that NAFLD is a strong independent predictor of cardiovascular disease and may play a central role in the cardiovascular risk of MS. When NAFLD is diagnosed, the person's overall cardiovascular risk factor profile should be reviewed to ensure that risk factors are being appropriately modified.

## Figures and Tables

**Table 1:** Characteristics of studies include in meta-analysis.

First author (year)	Country	Participants		Study design	Followup (person-year)	NOS score	Outcome measures
		No. in analysis	Characteristics				
Assy (2010)	Israel	800	Individuals with law to intermediate risk for CAD and presence of fatty liver	Cross-sectional	NA	7	Coronary heart disease coronary atherosclerosis coronary plague (%)

Choi (2008)	Korea	659	Healthy people	Cross-sectional	NA	7	Carotid atherosclerosis

Targher (2007)	Italy	2392	Diabetic patients	Cross-sectional	NA	7	Coronary heart disease cerebrovascular disease peripheral vascular disease

Hamaguchi (2012)	Japan	1647	Healthy people	Cohort	7115	8	Coronary heart disease ischemic stroke cerebral hemorrhage

Targher (2006)	Italy	800	Diabetic patients	Cross-sectional	NA	7	Coronary disease peripheral disease cerebral disease

Targher (2005)	Italy	744	Diabetic patients	Cohort	3720	7	Myocardial infarction coronary artery bypass grafting ischemic stroke cardiovascular events death


NOS score = Newcastle-Ottawa scale: used for quality assessment. We assigned NOS scores of 1–3, 4–6, and 7–9 for low, intermediate, and high-quality studies, respectively. These six studies are all adjusted for confounders, such as age, sex, diabetes duration, HbA1c, smoking history, LDL cholesterol, GGT levels and use of medications (i.e., hypoglycemic, antihypertensive, lipid-lowering, or anti-platelet drugs), and NCEP ATP III-defined MS.
